# Integrating eye care into Community Health Centers: a framework for advancing vision equity in underserved communities

**DOI:** 10.3389/frhs.2025.1697969

**Published:** 2025-12-18

**Authors:** Angelica C. Scanzera, Diane Russo, Susan A. Primo, Judes Fleurimont, Justin H. Markowski

**Affiliations:** 1 Department of Ophthalmology and Visual Sciences, Illinois Eye and Ear Infirmary, University of Illinois Chicago, Chicago, IL, United States; 2 Department of Clinical Education and Clinical Sciences, New England College of Optometry, Boston, MA, United States; 3 Department of Ophthalmology, Emory University School of Medicine, Atlanta, GA, United States; 4 Mile Square Health Center, Chicago, IL, United States; 5 Division of Health Policy and Administration, School of Public Health, University of Illinois Chicago, Chicago, IL, United States

**Keywords:** community health center, federally qualified health center, health equity, implementation framework, access to eye care, optometry, ophthalmology

## Abstract

Vision health is a critical yet often overlooked component of comprehensive primary care, particularly for underserved populations. Patient access to eye care services enhances workplace productivity, household income, and employment opportunities, ultimately supporting economic growth, poverty reduction, and food security**.** Community Health Centers (CHC) collectively serve over 32 million patients annually and are uniquely positioned to address disparities in eye care access. Yet only 26% of CHCs offer vision care services, and only 2.9% of people who access CHC services receive eye care. Addressing this gap requires a strategic, systems-level approach to implementation. This perspective proposes an integrated framework to guide the sustainable and equitable integration of eye care providers, including optometrists and ophthalmologists, into Community Health Centers (CHCs). Drawing on and uniting the Consolidated Framework for Implementation Research (CFIR), the National Institute on Minority Health and Health Disparities (NIMHD) Research Framework, and the National Association of Community Health Centers' (NACHC) Value Transformation Framework (VTF), we outline a multi-level strategy that addresses implementation readiness, equity, and sustainability. This integrated framework is intended to inform implementation research and policy development aimed at making on-site eye care via an optometrist or ophthalmologist a mandated service in CHCs nationwide. In doing so, we offer an actionable game plan for CHC leaders, healthcare administrators, and public health advocates to expand access to comprehensive eye care in underserved communities.

## Introduction

Eye health and vision loss are critical public health issues that disproportionately affect underserved populations (1, 2), including those served by Community Health Centers (CHCs). Despite national initiatives such as *Healthy People 2030*, access to eye care remains limited for rural, racially and ethnically minoritized, and low-income communities (2–5). As a result, conditions such as glaucoma**,** diabetic retinopathy, and uncorrected refractive error are more prevalent in these groups (6–10), with Black patients experiencing blindness rates two to four times higher than their White counterparts (7, 8).

Community Health Centers (CHC), which include Federally Qualified Health Centers that receive federal funding under Section 330 of the Public Health Service Act (11), are designed to address health disparities by providing comprehensive, community-based primary care to medically underserved populations. CHCs collectively serve over 32 million patients annually (12), providing essential services including primary and pediatric care, behavioral health, dental, pharmacy, substance use counseling, and immunizations, among others (13). This makes them uniquely positioned to address disparities in eye care access. Given the broad and compelling evidence documenting the value of CHCs, CHCs have traditionally received significant bipartisan support for their efforts (14–20).

Despite their mission, most CHCs do not offer on-site eye care services. The proportion of CHCs with vision care services decreased from 32% to 26% between 2021 and 2023, and only 2.9% of patients receiving care in CHCs received any form of eye care in 2023 (21–24). Barriers to integrating eye care services into CHCs include the need for assistance with creating a business plan for understanding billing and reimbursement, designing the space, and compiling an inventory of eye care equipment (21). Systemic barriers to providing vision care at CHCs include a shortage of eye care clinicians, limited funding and space for equipment, and complex implementation processes (25). Inadequate or complex reimbursement, competing health priorities, and low patient follow-up rates further hinder sustainable vision services (25, 26). However, systemic approaches to identifying, addressing, categorizing, and processing these implementation components have yet to be developed.

This represents a significant missed opportunity to address preventable vision loss and improve chronic disease management through integrated care. For example, CHC patients with diabetes are more likely to follow through with eye exams when referred by their primary care provider (27), underscoring the importance of integrated care. Furthermore, Diabetes and Diabetes prevention-focused programs already exist at many CHCs (28–31). Supplementing those programs with eye care integration components can offer a sustainable, cohesive alignment opportunity. Evidence also suggests that screening for refractive error and early eye disease could prevent a high proportion of unnecessary vision loss or blindness (32, 33). As CHCs care for one in three persons in poverty, one in six Medicaid enrollees, one in five uninsured persons, and one in five rural residents in the United States, improvements and expansion of services in CHCs will directly benefit thousands of underserved communities nationwide (34).

As the prevalence of common eye diseases continues to rise, it is critical to understand how to better integrate eye care providers into comprehensive ambulatory safety net settings. There is growing support among major health agencies and funders for implementation science (IS) to improve the adoption of evidence-based care, including eye care in CHCs (35–44). To address persistent barriers, such as organizational readiness, population health needs, and financial sustainability, a structured, theory-informed approach is essential.

This Perspective introduces a structured, multi-level framework to guide the integration of eye care providers into CHCs. Drawing on the Consolidated Framework for Implementation Research (CFIR), the National Institute on Minority Health and Health Disparities (NIMHD) Research Framework, and the National Association of Community Health Centers' (NACHC) Value Transformation Framework (VTF), we develop and outline a five-stage model that supports readiness assessment, service design, implementation, evaluation, and scale-up. This framework is intended to detail how CHCs can support and promote public health by offering a practical roadmap for expanding vision care access in safety net settings.

## An integrated framework to guide eye care provider integration into community health centers

To address the complex and multi-level barriers to integrating on-site eye care into CHCs, we propose a unified implementation framework that draws on three complementary models: CFIR (45, 46), the NIMHD Research Framework (47), and the VTF (48). Together, these frameworks provide a structured, comprehensive approach to understanding and guiding implementation efforts. CFIR offers a structured approach to assessing readiness in the implementation process; the NIMHD Research Framework embeds equity across all levels of influence; and VTF ensures alignment with value-based care and operational sustainability. This integrated framework supports the design, implementation, and scale-up of sustainable eye care services in CHCs.

1. **Consolidated Framework for Implementation Research (CFIR)** (45, 46, 49). CFIR provides tools to assess organizational readiness, identify implementation barriers, and guide adoption. It includes five domains: outer setting, inner setting, characteristics of individuals, innovation, and implementation process. This can help CHCs evaluate their internal and external environments, staff capacity, and integration strategies.
2. **National Institute on Minority Health and Health Disparities (NIMHD) Research Framework** (47). NIMHD describes an individual's health outcomes involving multiple levels of influence over the life course (47). The Research Framework created by NIMHD provides a multidimensional model that depicts a comprehensive set of health determinants, including domains of influence over the life course (biological, behavioral, physical/built environment, sociocultural environment, and the health care system) and levels of influence (individual, interpersonal, community, and societal) (47). This framework ensures that implementation efforts are equity-informed and responsive to the Social Determinants of Health (SDOH). Applying this framework ensures that eye care integration addresses disparities in access, cultural relevance, and systemic barriers to reduce health disparities through multidisciplinary research and community involvement.
3. **Value Transformation Framework (VTF)** (48)**.** Uniquely developed by the NACHC, the VTF supports CHCs in improving care delivery by aligning infrastructure, care delivery, and workforce with evidence-based strategies. With 15 change areas within the above 3 domains, it offers actionable ways to promote and sustain systems change, specifically focused on CHC improvement (48). VTF allows for aligning eye care integration within the broader goals of CHCs to provide quality and patient-centered care. It also supports the development of sustainable business models, including referral systems, telehealth capacity, and workforce development.

### Framework summary

Together, CFIR, the NIMHD Research Framework, and VTF form a multi-dimensional framework to guide the integration of eye care in CHCs. CFIR helps to identify and address organizational and process-level barriers and facilitators that influence implementation, the NIMHD Research Framework ensures the integration efforts are equity-informed and community-focused, and VTF supports the financial and operational sustainability of eye care services within CHCs. Table 1 summarizes each framework's focus and application to the goal, as well as key questions to consider.

**Table 1:** Summary of framework focus, level, application to goal, and key questions to consider.

Summary of framework			
Framework	Focus	Level	Application to goal
CFIR	Barriers & Facilitators Organizational readiness	Multi-level (individual, organizational, broader policy), implementation process	Identifies how to implement effectively within CHCs
NIMHD Research Framework	Health disparities Social determinants Equity	Multi-level (individual to societal)	Ensures patient- and community-level focus addressing SDOH & equity
VTF	Financial & operational sustainability Value based care Quality improvement Workflows Workforce development	CHC-system level	Ensures evaluation of quadruple aim (health outcomes, patient & staff experience, reduced costs.
Key Questions to consider in each stage			
Stage of implementation	Key questions		
Contextual Inquiry	CFIR: What level of support is needed from the CHC leadership to implement on-site eye care? What kinds of legislation, regulations, professional group recommendations, or accreditation standards might help or hinder implementation on-site eye care]? NIMHD: What individual and community-level social determinants of health and disparities exist in the community that may affect access to eye care? VTF: What infrastructure (e.g., space, technology, workflows) is currently in place to support on-site eye care?		
Implementation Planning	CFIR: To what extent is an implementation plan being developed? What does the plan include? To what extent are: roles and responsibilities being outlined? Specific steps and milestones being outlined? Implementation goals being set? NIMHD: What community input is needed in the design of this service and how do we best engage to build strong relationships? VTF: What resources (e.g., funding, equipment, staffing) are needed to operationalize on-site eye care, and how can they be secured? What health information technology can be leveraged to measure outcomes?		
Implementation	CFIR: To what extent is on-site eye care being implemented using small steps, tests, or cycles of change? NIMHD: How are we addressing individual and community-level barriers to care (e.g., transportation, child care, social support)? VTF: How are care delivery processes being adapted to integrate on-site eye care into existing workflows?		
Pilot Testing & Evaluation	CFIR: What information or data is being collected? Who is reviewing and discussing it? When or how frequently? What impact did on-site eye care have on CHC patients? NIMHD: Are there measurable improvements in preventable vision loss, health equity, or access to eye care among the CHC population? VTF: What are the direct and indirect cost implications of on-site eye care in the pilot? Has offering on-site eye care improved patient and/or staff satisfaction?		
Scale-Up	CFIR: How do you define successful adoption? What adaptations are needed to scale the model across different CHC contexts? NIMHD: How can we ensure that scale-up efforts continue to address disparities and promote equity? VTF: What financing or policy strategies can support long-term integration of eye care?		

*CFIR questions adapted directly from revised interview guide (49).

### Integration process

To develop this model, we systematically mapped each framework's domains to the five stages of implementation. CFIR's inner and outer settings guided organizational and community readiness assessments, while its innovation and process domains informed workflow design and fidelity monitoring. NIMHD's multi-level emphasis on social determinants and equity was layered across all stages to ensure interventions addressed disparities at individual, community, and societal levels. VTF contributed operational and financial sustainability elements, including infrastructure, cost analysis, and alignment with value-based care. This mapping process allowed us to identify complementary strengths and reduce redundancy, resulting in a cohesive, multi-level roadmap for CHCs.

**Figure 1:**
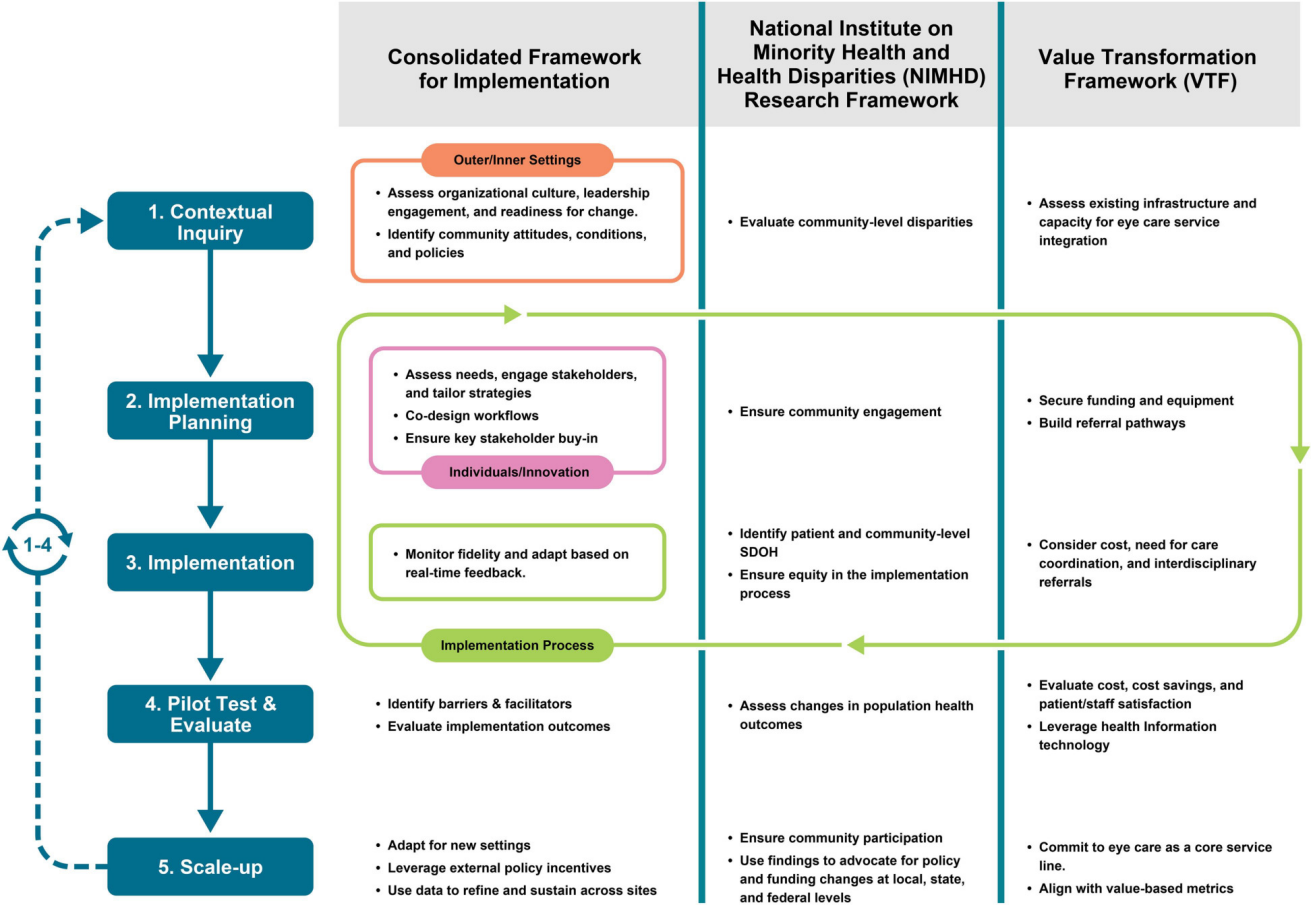
Integrated framework for eye care integration in community health centers (CHCs), combining CFIR, NIMHD research framework, and value transformation framework (VTF). The model maps complementary domains from each framework across five stages of implementation: contextual inquiry, implementation planning, implementation, pilot testing and evaluation, and scale-up. CFIR informs readiness and process evaluation; NIMHD embeds equity and addresses social determinants of health; VTF ensures operational sustainability and alignment with value-based care.

This integrated model works across five stages. **1)** Contextual inquiry: involves evaluating organizational readiness (CFIR), infrastructure (VTF), and community-level disparities (NIMHD); **2)** Implementation planning: focuses on co-designing service models, securing resources (e.g., space, equipment), building referral systems (i.e., surgical intervention), and using stakeholder/community input; **3)** Implementation: strategies are focused on addressing individual and community-level SDOH. Here, there is significant emphasis on stakeholder engagement and staff buy-in; **4)** Pilot testing and evaluation: use CFIR & Proctor's taxonomy to evaluate implementation outcomes, VTF to measure cost and care delivery, and NIMHD to assess population health outcomes; **5)** Scale-up: involves expanding eye care services by aligning with sustainable long term financial models, and advocating for policy changes to support adoption. This model (Figure 1) provides a roadmap for CHCs to implement eye care services that are sustainable, equitable, and scalable. Table 1 also shares questions to consider in each stage.

## Outcome evaluation

Evaluating the integration of eye care providers into CHCs requires a multi-dimensional approach that captures implementation, population health, and operational outcomes. This section emphasizes how the synthesis of CFIR, NIMHD Research framework, and VTF supports comprehensive outcome assessment across three domains. The integrated framework enables CHCs to assess fidelity, equity impact, and sustainability in a unified manner.

### Implementation outcomes

While the CFIR framework is instrumental in identifying barriers and facilitators to implementation, it does not directly measure implementation outcomes. Thus, we also incorporate Proctor's taxonomy to assess key implementation metrics (50). These include qualitative outcomes (e.g., acceptability, adoption, appropriateness, and fidelity) and quantitative outcomes (e.g., adoption, feasibility, penetration) (50) to assess how well on-site eye care services are integrated within workflows.

### Population health outcomes

The NIMHD Research Framework supports the evaluation of disparities in access, utilization, and outcomes at individual, community, and societal levels. Effectiveness indicators include rates of comprehensive eye exams among high-risk populations, reductions in preventable vision loss through early detection and treatment of conditions such as diabetic retinopathy, glaucoma, and amblyopia, as well as improvements in chronic disease management through coordinated care.

### Operational outcomes

VTF emphasizes outcomes related to care quality, cost-effectiveness, and patient experience. Metrics may include Medicaid/Medicare revenue, improvement in diabetes management, or reduction in emergency department visits. These outcomes demonstrate the clinical and financial value of integrating eye care in individual CHCs.

Together, these outcome domains reflect the strength of the integrated framework in supporting sustainable, equitable, and scalable eye care delivery in safety net settings.

## Practice and Policy Implications

This integrated framework provides a practical blueprint for implementing and sustaining eye care services in CHCs. It equips CHC leadership with tools to assess organizational readiness, identify and engage key stakeholders, and tailor implementation strategies to local contexts. The framework emphasizes equity by addressing patient and community-level social determinants of health (SDOH). It also supports alignment with quality improvement initiatives and value-based care, promoting a financially sustainable model for eye care delivery in CHCs.

Policy makers can also use this framework in guiding reimbursement reform and policy development. For example, expansion of Medicaid coverage for comprehensive eye care would directly support broader implementation efforts. While current research on the U.S. Medicaid program does not specifically examine eye care, its findings on improved access and reduced disparities following Medicaid expansion help set the stage for examining how similar changes could benefit comprehensive eye care services (51). A federal mandate requiring vision services as a component of primary care in CHCs, similar to dental or behavioral health (13), could also improve access and sustainability. Funders and researchers can support this transformation by investing in pilot programs, implementation research, and workforce development initiatives to build capacity in CHCs.

### Applying the framework in a Community Health Center: A theoretical use case

To illustrate the practical application of this framework, consider a theoretical example of an urban CHC serving a predominantly low-income population with high rates of diabetes and limited access to specialty care.

#### Stage 1: Contextual inquiry

Organizational readiness is assessed by external designers and implementation researchers using a CFIR-based survey and semi-structured interviews with CHC leadership, frontline staff, the quality improvement team, and the Community Advisory Board. These tools are publicly available and evaluate leadership engagement, available resources, and readiness for change {Research, 2023 #1337}. Strong leadership support and space are identified and it is also determined that there is currently limited staffing. A community health needs assessment (CHNA), required to be conducted every 3 years by FQHCs, is conducted by the CHC's research team and included questions about history of eye disease and whether individuals have had an eye exam in the prior 12 months (52). This CHNA reveals high rates of diabetic retinopathy in the community compared to national and county prevalence rates as estimated by the CDC {CDC, 2025 #1577} {Lundeen, 2023 #1578}, as well as low rates of diabetic eye examinations in CHC patients, a Healthcare Effectiveness Data and Information Set (HEDIS) measure (53). Additionally, internal assessment finds gaps in care coordination as well as billing.

#### Stage 2: Implementation planning

Findings from contextual inquiry directly inform strategy selection. The CHC forms a multidisciplinary planning team, including primary care, finance, quality improvement, information technology, frontline staff, and CHC patients. A service model is co-designed to include part-time on-site optometry services, the addition of an AI-based diabetic retinopathy screening using fundus camera operated by trained image takers, and a referral pathway to ophthalmology for individuals requiring treatment for diabetic retinopathy. This approach ensures comprehensive coverage across the CHC network while addressing workforce constraints. Pilot funding is secured through CHC partnerships to support equipment purchase and optometrist staffing. Additional community input is gathered through meetings with the Community Advisory Board and interactive workshops to ensure cultural relevance and build community trust. Specific questions and considerations are included in Table 1.

#### Stage 3: Implementation

Implementation is explicitly tied to barriers identified during contextual inquiry and the CHC launches a pilot at one site. Standing orders for diabetic eye exams are introduced to reduce missed referrals, addressing gaps in care coordination. Confidence and fidelity are built through staff training that incorporates practice based learning on the AI-based diabetic retinopathy screening camera, as well as protocols for referrals to the internal optometrist and external ophthalmologist based on screening results. Community health workers assist patients with scheduling and transportation, mitigating social determinants of health that may have been identified during contextual inquiry. Standing meetings are held to monitor fidelity, review early data, and enable rapid prototyping and adaptation of workflows.

#### Stage 4: Evaluation

Implementation outcomes are evaluated using Proctor's taxonomy and include adoption, fidelity, and acceptability. Adoption is measured as the percentage of eligible patients with diabetes screened using electronic health record data, fidelity is assessed through workflow reviews, and acceptability is evaluated via staff and patient surveys. Several measurement resources are publicly available through the Dissemination & Implementation Models in Health Research and Practice Webtool {University of Colorado Denver, 2025 #1579}. Cost-effectiveness is analyzed by comparing implementation costs to revenue generated, which includes reviewing billing codes used by the optometrist for comprehensive eye exams and procedures, as well as reimbursement rates for AI-based diabetic retinopathy screening. Population health metrics include changes in CHC-level diabetic eye exam rates in comparison to community-level metrics, as well as rates of referrals for treatment.

#### Stage 5: Scale-up

Upon positive pilot results, the CHC creates the program as a permanent service and expands its screening to additional CHCs. The CHC then begins to evaluate screening for glaucoma and refractive error. At the state level, partners begin advocating for policy changes to support on-site vision care statewide using data gathered from the pilot.

## Discussion

Despite the high burden of vision impairment and the availability of effective interventions, most CHCs lack integrated eye care services. This Perspective responds to a long-standing gap in primary care by offering a structured, equity-informed framework to guide the integration of optometrists and ophthalmologists into CHCs. By combining CFIR, NIMHD, and VTF, the model addresses readiness, equity, and sustainability, three critical dimensions often overlooked in specialty care integration.

The integrated framework offers a practical roadmap for CHC leaders, policymakers, and public health stakeholders to implement and sustain eye care services in underserved settings. However, successful implementation will require overcoming persistent challenges, including limited space, staffing shortages, and reimbursement constraints. CHCs vary widely in infrastructure and capacity, and adaptation of this framework must be context-specific. Stakeholder engagement, particularly with CHC administrators, eye care providers, and patients, will be essential to ensure feasibility and community buy-in. This framework outlines a practical playbook for CHC leaders and policymakers to support the sustainable and equitable expansion of access to these essential services in underserved communities across the US.

While conceptual, this framework is grounded in evidence-based models and informed by the operational realities of CHCs. It serves as a critical starting point for researchers, providers, and policymakers to build and test implementation strategies that are contextually relevant, equity-focused, and scalable. Ultimately, this integrated framework supports a long-term policy goal, the inclusion of on-site eye care services by an eye care provider as a required component of primary care in CHCs. With coordinated advocacy, research, and investment, it has the potential to transform vision care delivery and reduce preventable blindness in underserved communities.

## Data Availability

The original contributions presented in the study are included in the article/Supplementary Material, further inquiries can be directed to the corresponding author.
